# Carotid intima-media thickness is an independent predictor of all-cause mortality and cardiovascular morbidity in patients with diabetes mellitus type 2 and chronic kidney disease

**DOI:** 10.1080/0886022X.2019.1585372

**Published:** 2019-03-26

**Authors:** Athanasios Roumeliotis, Stefanos Roumeliotis, Stylianos Panagoutsos, Marios Theodoridis, Christos Argyriou, Anna Tavridou, George S. Georgiadis

**Affiliations:** a Department of Nephrology, “Democritus” University of Thrace, Medical School, University General Hospital of Alexandroupolis, Alexandroupolis, Greece;; b Department of Vascular Surgery, “Democritus” University of Thrace, Medical School, University General Hospital of Alexandroupolis, Alexandroupolis, Greece;; c Laboratory of Pharmacology, “Democritus” University of Thrace, Medical School, Alexandroupolis, Greece

**Keywords:** Carotid intima-media thickness, diabetes mellitus type 2, cardiovascular disease

## Abstract

**Background:** Intima-Media-Thickness of the carotid artery wall (cIMT) is a strong predictor of cardiovascular (CV) disease. The aim of this study was to investigate the significance of cIMT as an independent prognostic factor for CV morbidity and mortality in patients with chronic kidney disease (CKD) and diabetes mellitus type 2 (DM2).

**Methods:** The study included 142 diabetic patients in different stages of CKD. Patients were categorized into two groups according to low (≤0.86 mm) or high cIMT (>0.86 mm), respectively. CV events and death from all causes were registered during a seven-year follow-up.

**Results:** Mean age, BMI and duration of diabetes were 68 years (range: 45–90), >30 kg/m^2^ and 15 years (range: 5–40), respectively. Patients with increased cIMT were older, suffered from a lower estimated glomerular filtration rate (eGFR), peripheral atherosclerosis and plaque presence in either carotid artery. Increased BMI (beta= −0.29, *p* = .01), lower eGFR (beta = 0.353, *p* = .003) and male gender (beta= −0.339, *p* = .005) were found to predict increased cIMT. Predictors of all-cause mortality in Cox proportional hazard models were low eGFR and high cIMT with HR = 0.96 (CI = 0.94–0.98), *p* < .001 and HR = 2.9 (CI = 1.03–7.99), *p* = .04, respectively. The risk of future CV event was determined by albuminuria and cIMT with HR = 1 (CI = 1.0–1.0), *p* < .001 and HR = 2.04 (CI = 1.1–3.78), *p* = .02, respectively. Patients with high cIMT presented significantly higher all-cause mortality and a new CV event (*p* = .005/*p* = .018, respectively).

**Conclusions:** cIMT is a strong and independent predictor of CV morbidity and mortality, and should be considered a valuable tool for the stratification of CV risk in patients with CKD and DM2.

## Introduction

Patients with chronic kidney disease (CKD) and diabetes mellitus type 2 (DM2) are considered to have an increased risk of developing cardiovascular (CV) complications [1,2]. In particular, CKD patients have a 2-fold higher incidence of CV events when compared to the general population [2] whereas diabetes mellitus is also considered to be a coronary artery disease equivalent in terms of clinical significance [3]. Therefore, patients suffering from concurrent CKD and DM2 are more likely to die of CV cause than to progress to end-stage renal disease (ESRD) [4]. The most important risk factor leading to CV morbidity and mortality in patients with DM2 and CKD is atherosclerosis.

Risk factors associated with the development of future atherosclerotic CV include older age, hypertension (HTN) and higher body mass index (BMI) [5]. A noninvasive surrogate marker of subclinical atherosclerosis in the general population [6] is considered to be carotid intima-media thickness (cIMT), which has been associated with the presence of coronary artery disease and stroke [7]. cIMT has been correlated with future CV events [8] and mortality among different subpopulations, including hemodialysis (HD) patients [9] and patients with DM [10]. Although cIMT has been thoroughly studied in HD patients and healthy individuals, data investigating the association and predictive value between cIMT of diabetic patients in progressing stages of nephropathy with CV morbidity and mortality are lacking.

The aim of this study was to investigate whether cIMT is strongly correlated with increased CV morbidity and mortality in patients with DM2 in different stages of CKD.

## Subjects and methods

### Study population

Among the 346 patients initially enrolled, 181 were excluded because they did not suffer from diabetic nephropathy, as they only had diabetes and nephropathy, but without albuminuria. From the rest 165 patients of Greek Caucasian origin followed at the Diabetic Nephropathy Clinic of our hospital, 142 patients were included in the study and an informed consent was obtained. Patients included suffered for at least 5 years from DM2, as defined by the American Diabetes Society [11], diabetic retinopathy (DR) and nephropathy. DR was included in the eligibility criteria, because its presence with nephropathy in the diabetic population is frequent as well as indicative of nephropathy. Patients were considered to suffer from DR if they had undergone retinal laser treatment (photocoagulation) and/or after fundoscopy assessment following papillary dilatation. Established diabetic nephropathy was defined by persistent albuminuria presence in at least two out of three consecutive measurements in sterile spot morning urine over a 6-month period, as described elsewhere [12]. The diagnosis and classification of CKD stages were performed according to the Clinical Practice Guidelines for Chronic Kidney Disease from the National Kidney Foundation-Kidney Disease Outcomes Quality Initiative [13]. Patients with diabetes duration less than 5 years, no signs of DR, absence of albuminuria and nephropathy, diagnosis of obstructive uropathy, active cancer or acute illness were excluded from the study.

The included population consisted of 77 male and 65 female diabetic patients with a mean age of 68 years (range 45–90), mean DM2 duration of 15 years (range 5–14), hypertension and DR. According to CKD stages, 38 (26.8%) of them were in stage 1, 18 (12.7%) in stage 2, 32 (22.5%) in stage 3, 30 (21.1%) in stage 4 and 24 (16.9%) in stage 5. Demographic data, clinical characteristics, history of a previous CV event, blood sampling results and measurement of cIMT parameters were recorded at baseline. All participants were followed over a period of seven years, from November 2008 through November 2015. The end-points studied were all-cause mortality and CV morbidity and mortality. CV events were defined as myocardial infarction (MI), stroke or peripheral arterial disease (PAD) and were documented by the according specialist, blinded to the study results. A patient was deemed to suffer from PAD if they suffered from claudication, acute limb ischemia, gangrene or skin ulcers related to ischemia. Patient follow up was performed through regular visits at the clinic, from death certificates or via an integrated telephone interview. Our study was approved by the Scientific Council and the Ethics Committee according to the Helsinki Declaration of Human Rights.

### Laboratory methods

Blood samples were drawn after an overnight fast of at least 10 h and were consequently assessed for biochemical parameters (creatinine, fasting glucose, glycosylated hemoglobin (HbA1c), serum lipids) and albuminuria.

The presence of albuminuria was evaluated by the measurement of albumin (turbidimetric immunoassay) and creatinine in a morning spot urine collection. Albuminuria was calculated in milligrams (mg) of albumin per gram of creatinine in the urine (UACR, mg/gram). EGFR (estimated glomerular filtration rate) estimation was made through the chronic kidney disease epidemiology collaboration (CKD-EPI) equation as described elsewhere [14].

### Carotid ultrasonography

CIMT was measured in every patient using a high-resolution, B-mode, real-time ultrasonography with a 7.5 MHz transducer (ATL Ultrasound HDI 1300, Phillips, Bothell., WA) by a single well-trained, validated examiner. CIMT was defined as the distance between the leading (echogenic) edge of the luminal-intimal interface and the leading (echogenic) edge of the media-adventitia interface, according to the American College of Cardiology [15,16]. Three cIMT values were obtained in three sites, respectively which included the common carotid artery, the carotid bulb (1 cm distally) and the internal carotid artery. The values were measured within a 1 cm length at each site. The mean of 18 repetitive measurements was documented (nine for each carotid artery) and defined as mean cIMT. Maximum cIMT was defined as the mean of the two maximum values, one on each carotid artery. Carotid plaque was defined as a focal extrusion into the arterial lumen at least 0.5 mm or 50% of the surrounding cIMT value, or a thickness of >1.5 mm. Our study population was subsequently divided into two groups based on the estimated median cIMT value: those with cIMT value above 0.86 mm and those with below or equal 0.86 mm. The percentage of patients with an increased cIMT (>0.86) according to CKD stage was: 31.6% in stage 1, 44.4% in stage 2, 65.6% in stage 3, 33.3% in stage 4 and 47.9% in stage 5 patients, respectively.

### Statistical analysis

Statistical analyses were performed using the IBM Statistical Package for Social Sciences (SPSS 18.0 for Windows, Chicago, IL, USA). Data were tested for normality by Kolmogorov–Smirnov test and are presented as mean ± SD, median and range or percentage, as appropriate. Study parameters were compared between patients with high (>0.86 mm) and low (≤0.86 mm) cIMT using a chi-square test for categorical variables and analysis of variance (ANOVA) for continuous variables. For bivariate associations between variables, we used Spearman’s correlation coefficient.

Stepwise forward regression analysis was conducted with the natural logarithm of cIMT (cIMTln) as the dependent variable with classic atherosclerotic risk factors (age, gender, eGFR, BMI, systolic and diastolic blood pressure, smoking, total, LDL- and HDL-cholesterol, triglycerides, CRP, HbA1C, UACR and DM2/dyslipidemia/hypertension duration) on an exploratory basis to determine possible predictors of cIMT. We also examined possible predictors for the presence of an atherosclerotic plaque in either carotid artery.

For survival analysis, all patients were categorized into two groups according to the median value of cIMT (>0.86 or ≤0.86 mm). Survival analyses included Kaplan–Meier curves and log-rank tests. We used multivariate Cox proportional hazard models (forward stepwise selection) to calculate adjusted hazard ratios, and 95% CIs for all-cause mortality and CV events. The model was adjusted for various potential confounding factors (age, sex, eGFR, BMI, systolic/diastolic blood pressure, duration of hypertension, duration of DM2, total-, LDL- and HDL-cholesterol, triglycerides, CRP, HBA1c, albuminuria and cIMT). Statistical significance was reached at a *p* values <.05.

## Results

Among 165 patients initially presented, 142 patients were finally included and categorized depending on the cIMT median value (high > 0.86 or low ≤ 0.86 mm). The demographic/clinical and biochemical characteristics of the included population are shown in Table 1 and Table 2, respectively. The mean age of the patients was 68 years (range 45–90), 54% of whom were men. All the patients presented with DM2, hypertension and diabetic retinopathy. At baseline, patients in the high cIMT group versus those in the low were significantly older (70 ± 9 vs. 67 ± 9 years old respectively, *p* = .04), with worse kidney function (41.3 vs. 53.4 mL/min/1.73 m^2^, *p* = .023), more likely to suffer from PAD (58.8% vs. 31.1%, *p* = .001) and to have advanced atherosclerosis in either carotid artery (82.3% vs. 52.7%, *p* < .001). There was no significant difference found between the cIMT groups regarding DM2, HTN and dyslipidemia duration, as well as the class of anti-diabetic and anti-hypertensive drugs they were receiving.

**Table 1.** Demographic and clinical parameters of patients with type 2 diabetes and diabetic retinopathy with cIMT above and below median value (mm).

	All subjects	cIMT ≤0.86 mm	cIMT >0.86 mm	*p*
*N*	142	74	68	
Age (years)	68 ± 9	67 ± 9	70 ± 9	.04
Gender (male/female)	77/65	36/38	41/27	.11
BMI (kg/m^2^)	30.7 ± 5.2	30.1 ± 5.1	31.4 ± 5.3	.13
Hypertension duration (years)	15 ± 8	14 ± 8	15 ± 7	.32
DM2 duration (years)	15 ± 8	15 ± 8	16 ± 7	.34
Dyslipidemia presence (%)	123 (86.6%)	62 (89.7%)	61 (89.7%)	.30
Dyslipidemia duration (years)	8 ± 5	7 ± 5	8 ± 6	.32
Smoking (y, %)	30 (21.1%)	13 (17.6%)	17 (25%)	.19
Stroke (y, %)	18 (12.7%)	8 (10.8%)	10 (14.7%)	.33
MI (y, %)	46 (32.4%)	20 (27.0%)	26 (38.2%)	.11
PAD (y, %)	63(44.4%)	23 (31.1%)	40 (58.8%)	.001

*p* values of chi-square test or one-way ANOVA for differences of variables among patients with cIMT above or below 0.86 mm.

Results for continuous variables are presented as mean (S.D) or median (range). CIMT: carotid Intima-Media-Thickness; BMI: Body Mass Index; DM2: Diabetes Mellitus type 2; MI: myocardial infarction; PAD: peripheral arterial disease.

**Table 2.** Biochemical parameters of patients with type 2 diabetes, CKD and diabetic retinopathy with cIMT above and below median value (mm).

	All subjects	cIMT ≤0.86 mm	cIMT >0.86 mm	*p*
*N*	142	74	68	
HbA1c (%)	7.5 ± 1.1	7.6 ± 1.1	7.4 ± 1.2	.27
SBP (mmHg)	137 ± 17	137 ± 17	138 ± 18	.721
DBP (mmHg)	77 ± 9	77 ± 9	78 ± 8	.513
Total cholesterol (mg/dl)	179.4 ± 52.5	177.6 ± 56.6	181.4 ± 48.1	.67
LDL-cholesterol (mg/dl)	101.8 ± 43.9	102.1 ± 48.3	101.6 ± 39.1	.95
HDL-cholesterol (mg/dl)	45.7 ± 12.7	46.6 ± 13.7	44.7 ± 11.5	.38
Triglycerides (mg/dl)	161 (27–966)	125 (27–966)	154 (52–450)	.51
UACR (mg/g)	664.9 ± 1751.3	780.4 ± 1967.6	471.5 ± 1428.5	.35
CRP (mg/dL)	0.71 ± 1.65	0.66 ± 1.53	0.76 ± 1.78	.71
eGFR	47.6 ± 31.8	53.4 ± 33.6	41.3 ± 28.7	.023
Mean cIMT (mm)	0.86 (0.40–1.50)	0.73 (0.40–0.86)	1.00 (0.87–1.50)	<.001
Maximum cIMT (mm)	0.95 (0.50–1.50)	0.80 (0.50–1.20)	1.10 (0.90–1.50)	<.001
Plaque presence (%)	95 (66.9%)	39 (52.7%)	56 (82.3%)	<.001

*p* values of chi-square test or one-way ANOVA for differences of variables among patients with cIMT above or below 0.86 mm.

Results for continuous variables are presented as mean (S.D) or median (range). CIMT: carotid Intima-Media-Thickness; HbA1c: hemoglobin A1c; SBP/DBP: systolic/diastolic blood pressure; LDL: Low-Density Lipoprotein; HDL: High-Density Lipoprotein; UACR: urine albumin to creatinine ratio; CRP: C-reactive protein; eGFR: estimated glomerular filtration rate.

Table 3 shows the mean (0.86 mm) and maximum cIMT (0.95 mm) correlation with demographic biochemical, and clinical data of the included population. At baseline, multivariate analysis showed that both mean cIMT and cIMT max were positively correlated with age (*r* = 0.217, *p* = .01 and *r* = 0.210, *p* = .012, respectively) and were both inversely associated with eGFR (*r*= −0.221, *p* = .008 and *r*= −0.185, *p* = .028, respectively). Again, multivariate analysis showed that mean values of cIMT were significantly associated with DM2 and hypertension duration (*r* = 0.170, *p* = .044 and *r* = 0.205, *p* = .014, respectively), while the duration of dyslipidemia was marginally not significantly associated (*r* = 0.163, *p* = .062). In the same analysis, maximum cIMT values were significantly associated with dyslipidemia duration (*r* = 0.183, *p* = .037) and marginally not statistically significant with DM2 and hypertension duration (*r* = 0.159, *p* = .06 and *r* = 0.163, *p* = .053, respectively). Both mean and maximum cIMT was not found statistically significant with BMI, waist circumference, total-, HDL- and LDL-cholesterol levels, HbA1c and albuminuria. On the other hand, mean cIMT was positively correlated with triglyceride levels (*r* = 0.176, *p* = .037), while maximum cIMT was marginally not significantly associated (*r* = 0.164, *p* = .053).

**Table 3.** Correlation matrix between cIMT and cIMT max with various risk factors.

	cIMT (mm)		cIMT max (mm)	
	*r*	*p*	*r*	*p*
Age	0.217^a^	.010	0.210^b^	.012
BMI (kg/m2)	0.128	.128	0.107	.206
Waist circumference (cm)	0.065	.441	0.031	.715
Duration of DM2 (years)	0.170^b^	.044	0.159	.06
Duration of Hypertension (years)	0.205^b^	.014	0.163	.053
Duration of dyslipidemia (years)	0.163	.062	0.183	.037
Systolic BP (mmHg)	0.059	.491	0.068	.422
Diastolic BP (mmHg)	0.030	.724	0.013	.879
Total chol (mg/dl)	0.024	.777	0.035	.678
TGs (mg/dl)	0.176^b^	.037	0.164	.053
HDL-chol (mg/dl)	−0.092	.281	−0.050	.554
LDL-chol (mg/dl)	−0.010	.911	−0.008	.926
CRP (mg/dl)	0.104	.222	0.084	.324
HbA1c (%)	−0.099	.251	−0.102	.263
Albuminuria, UACR (mg/g Crea)	0.080	.400	0.045	.633
eGFR (mL/min/1.73 m^2^)	−0.221^a^	.008	−0.185^b^	.028

^a^Correlation is significant at the 0.01 level (2-tailed).

^b^Correlation is significant at the 0.05 level (2-tailed).

Values represent Spearman’s correlation coefficients.

We also examined the presence of an atherosclerotic plaque in at least one carotid artery. Male patients were more likely to have a carotid plaque versus female patients (75.3% vs. 56.9%, respectively, *p* = .016). Factors such as eGFR, age, BMI, waist, systolic/diastolic blood pressure, HTN-, DM2-, dyslipidemia duration, prior CV event, albumin, lipid levels, CRP, HbA1c and albuminuria were not found to be significantly associated with carotid plaque presence (results not shown).

We performed stepwise forward multiple regression analysis to assess the impact of several risk factors on cIMT (Table 4). When adjusted for various well established confounders of atherosclerosis (age, sex, eGFR, BMI, systolic/diastolic blood pressure, HTN-, DM2-, dyslipidemia- duration, total, HDL-, LDL-cholesterol, triglycerides, HbA1c and UACR), only eGFR, BMI and gender remained significant predictors of cIMT (*p* = .001, .003 and .005, respectively).

**Table 4.** Stepwise forward multiple regression analysis with mean cIMTln as the dependent variable.

	Beta	Standard error	*p*
Model 1 (unadjusted)			
eGFR (mL/min/1.73 m^2^)	−0.339	0.075	.005
Model 2			
eGFR (mL/min/1.73 m^2^)	−0.031	0.001	.005
BMI	0.244	0.006	.037
Model 3			
eGFR (mL/min/1.73 m^2^)	−0.29	0.001	.01
BMI	0.353	0.006	.003
Gender	−0.339	0.058	.005

Models adjusted for eGFR, age, sex, BMI, waist, SBP, DBP, HTN-DM2-dyslipidemia duration, total, HDL-, LDL-cholesterol, triglycerides, CRP, HbA1c and albuminuria.

The follow up of the study population was 7 years. Data for all-cause mortality and CV morbidity were recorded. During the follow-up period, 44 patients (31%) died from all-cause mortality and 66 patients presented a new CV event – stroke, myocardial infarction or peripheral artery disease (46.5%). No dialysis event was noted during this period of time.

A Cox proportional hazards regression analysis model was performed for all-cause mortality and included age, sex, eGFR, BMI, systolic/diastolic blood pressure, total-, LDL- HDL- cholesterol, triglycerides, CRP, HBA1c, UACR and cIMT. In the multivariate model, independent prognostic value was found to be significant for the eGFR (*p* < .001) and the cIMT value (*p* = .04). Patients with cIMT > 0.86 mm exhibited an almost three-fold hazard ratio of death manifestation.

A Cox proportional hazards regression analysis model was also performed for new CV event presentation and included age, sex, eGFR, BMI, systolic/diastolic blood pressure, total-, LDL- HDL- cholesterol, triglycerides, CRP, HBA1c, UACR and cIMT. Statistically significant independent parameters in the multivariate analysis were only albuminuria (*p* < .001) and cIMT value (Table 5). Patients with high cIMT manifested a two-fold risk of presenting with a new CV event.

**Table 5.** Cox proportional hazard analysis (forward stepwise regression) showing predictors for (A) all-cause mortality and (B) cardiovascular events in univariate and multivariate models.

Adjusted model				
	B	HR	CI	*p*
(A) All-cause mortality				
eGFR (mL/min/1.73 m^2^)	−0.04	0.96	0.94–0.98	<.001
CIMT > 0.86 mm	1.05	2.9	1.03–7.99	.04
(B) Cardiovascular event				
UACR (mg/g Creatinine)	<0.001	1.00	1.00–1.00	<.001
CIMT > 0.86 mm	0.71	2.04	1.10–3.78	.02

Adjusted model: Multivariate model, adjusted for age, sex, eGFR, BMI, Systolic/Diastolic Blood pressure, total-, LDL- HDL- Cholesterol, Triglycerides, CRP, HBA1c albuminuria (UACR) and cIMT.

HR: Hazard Ratio; CI 95%: Confidence Interval.

**Figure 1.**
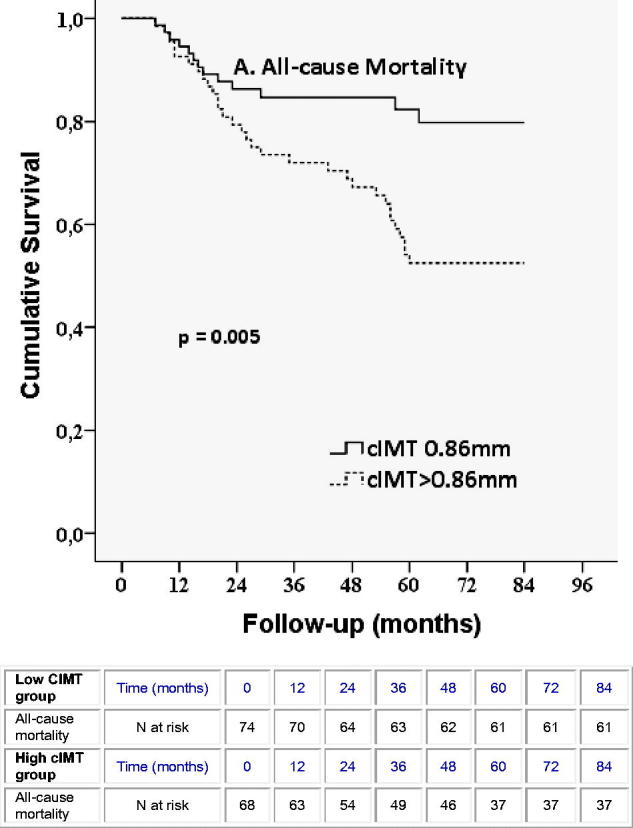
Kaplan–Meier curves for all-cause mortality in patients according to median cIMT value, below or above 0.86 mm.

The survival curve for all-cause mortality is shown in Figure 1. The two groups of patients, with high cIMT > 0.86 mm versus low cIMT ≤ 0.86 mm, differed significantly in their survival rates. In the low-cIMT group, mortality was 17.6% (13 out of 74 patients), while patients with high cIMT reached a mortality rate of 45.6% (31 out of 68 patients). Survival rates were statistically significant in the log-rank test (*p* = .005).

**Figure 2.**
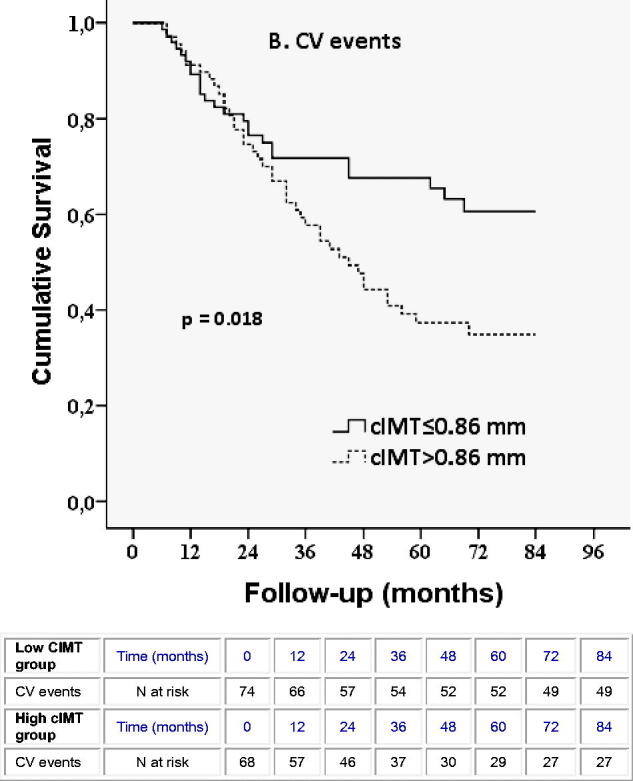
Kaplan–Meier curves for cardiovascular events in patients according to median cIMT value, below or above 0.86 mm.

The survival curve for the CV event manifestation is shown in Figure 2. Regarding the manifestation of new CV events, a statistically significant difference was observed between the two cIMT groups. Patients with cIMT > 0.86 exhibited a 60.3% CV event rate, while patients with low cIMT only 33.8% of any CV event. Differences were statistically significant in the log-rank test (*p* = .018).

## Discussion

Cardiovascular disease is the leading cause of death worldwide, especially in patients with risk factors such as diabetes and CKD [5,9,17]. As the underlying pathophysiologic mechanism is mainly atherosclerosis [18], CV risk assessment is crucial and could be performed through vascular imaging, taking into consideration atherosclerotic risk factors [9,19].

cIMT is increasingly used as a marker of atherosclerotic burden, but the ability to predict future CV events requires further investigation [5]. Furthermore, no consistent methods of measurement or standardized cIMT values exist in the current literature, rendering the whole issue open to debate [5]. The aim of our study was to evaluate the atherosclerotic burden by measuring cIMT values in a diabetic population in every stage of CKD as well as assessing cIMT as a clinical marker for the prediction of CV morbidity and mortality. To the best of our knowledge, this is the first study that correlates cIMT as an independent risk factor for the presence of CV disease in a population with DM2 in different CKD stages.

Most studies have focused either on the general population or the ESRD-hemodialysis patients in order to correlate cIMT with CV risk [9,17,18,20–23]. In our study, we included patients with DM2 in different CKD stages, who also suffered from hypertension and diabetic retinopathy, with a mean age of 68 years, ranging from 45 to 90 years. We divided them into two groups based on their median cIMT values (>0.86 mm and ≤0.86 mm). Our patients in both groups were relatively well controlled regarding their DM2 and HTN. They exhibited a mean HbA1c of 7.5%±1.1% and mean blood pressure values of 137 ± 17 mmHg (systolic) and 77 ± 9 mm Hg (diastolic).

At baseline, we found that patients with cIMT > 0.86 mm were older, suffered from worse kidney function, PAD and presence of carotid atherosclerotic plaque. The mean cIMT value of our study population (0.86 mm) was similar to values found in a study of Szeto et al., in which 203 Chinese patients with CKD stage 3–4 (0.81 mm) were included [20], in 900 outpatients with established atherosclerosis (0.90 mm) [22] and in a meta-analysis where mean cIMT ranged from 0.72 to 0.97 mm in the diabetic population [18]. They appeared, though, lower than values found in 5000 patients above 65 years of age without nephropathy (1.06–1.4 mm) [17], in 185 dialysis patients (1.02–1.05 mm) [9]. A study by Preston et al., showed that patients with CKD had higher mean cIMT values (0.59 mm) compared to healthy normotensive individuals (0.44 mm) [23].

As the exact cIMT measurement method remains controversial [24] we measured both mean and maximum cIMT in our patients in an effort to find a better outcome predictor. Both values were associated with older age and worsening kidney function. Mean cIMT (0.86 mm) was found to be positively correlated with duration of DM2 (*p* = .044) and HTN (*p* = .014), while dyslipidemia was marginally not correlated (*p* = .062). Maximum levels of cIMT (0.95 mm) on the contrary, were found to be positively correlated with dyslipidemia (*p* = .037), while marginally not correlated with duration of DM2 (*p* = .06) and HTN (*p* = .053). This slight difference may be attributed to the small number of patients enrolled. Both mean and maximum CIMT values were not correlated with BMI, waist circumference, HbA1c, cholesterol levels and albuminuria. In the multiple regression analysis, when adjusted for multiple confounders such as eGFR, age, sex, BMI, waist, SBP, DBP, hypertension-DM2-dyslipidemia duration, total, HDL-, LDL-cholesterol, triglycerides, CRP, HbA1c and albuminuria, we found that the only strong predictors of cIMT were age, male gender and BMI. Our study though, did not show any correlation between cIMT and cholesterol-total, LDL or HDL- levels.

At the 7-year follow-up period, 31% of the included patients (44 out of 142) died, whereas 46.5% (66 out of 142) of the study population presented with a new CV-event: stroke, myocardial infarction or peripheral artery disease. Additionally, the present study showed that the diabetic-CKD patients with the higher cIMT values (>0.86 mm) had a greater risk of death (hazard ratio of 2.9) or experiencing a new CV event (hazard ratio of 2) during the 7-year follow-up period. Many studies in the general population have documented the utility of cIMT in predicting CV morbidity and mortality with a 10–15% greater risk [5,17,25]. Regarding high-risk population such as the diabetics, cIMT confers an up to 2.2 times higher risk of CV morbidity and mortality than the general population [5,26]. CIMT can also strongly predict future CV events and mortality in CKD patients [5, 20], which was shown in CKD stages 3 and 4 [20] of dialysis patients with mortality hazard ratios from 1.2 up to as high as 3.2 [9,27,28]. However, we did not find any study strictly referring to patients with diabetes and nephropathy or correlating the predictive value of cIMT with CV morbidity and mortality.

In our Cox regression models, both higher cIMT and lower eGFR represented independent prognostic factors for shorter life expectancy. Additionally, higher cIMT and albuminuria levels conferred prognostic value to a new CV episode. Albuminuria, decreased eGFR, aging, hypertension, dyslipidemia, diabetes, and smoking in several populations such as the random population, CKD patients, diabetics and healthy individuals can independently predict CV morbidity and mortality as well as the progression of atherosclerosis [21,29–31]. Thus, we suggest that cIMT measurement should be included in the daily clinical practice of diabetic patients with CKD because the early stages of atherosclerosis could be detected in this high-risk population and timely intensive therapy regarding diabetes, hypertension, dyslipidemia and obesity could be initiated.

Although the clinical outcome is the ‘gold standard’ for evaluating the efficacy of a certain therapy, quantitative biomarkers such as HbA1c, serum lipids and blood pressure have been suggested as surrogate end-points to evaluate risk for death and new CV events. However, none of the current biomarkers is ideal. In our study population which consisted of diabetic, hypertensive patients with nephropathy and DR, cIMT measurement can be a good predictive marker for all-cause mortality and new CV event incidence.

The present study exhibits some limitations. Firstly, the study sample was relatively small. Furthermore, although we adjusted for all traditional CV risk factors in our analyses, CV morbidity and mortality may be influenced by other underlying confounding factors such as pro-inflammatory cytokines and endothelial dysfunction molecules. Thus, prospective studies of a larger sample should be conducted to verify our results.

## Conclusion

cIMT is an atherosclerotic marker with a wide applicability. There is no robust evidence to support that cIMT is strongly correlated with CV disease in the diabetic-CKD patient population. Our study shows that, cIMT has a positive predictive value for CV morbidity and mortality in our study population. The present study suggests that cIMT predictive value for the development or the presence of CV disease is significant, therefore, should be incorporated in the physician daily practice. However, further studies are needed in relation to traditional risk factors and high-risk populations.
